# Conservation genetics of two threatened frogs from the Mambilla highlands, Nigeria

**DOI:** 10.1371/journal.pone.0202010

**Published:** 2018-08-15

**Authors:** Denise Arroyo-Lambaer, Hazel Chapman, Marie Hale, David Blackburn

**Affiliations:** 1 Instituto de Biología, Universidad Nacional Autónoma de México, Ciudad Universitaria, Ciudad de México, México; 2 School of Biological Sciences, University of Canterbury, Christchurch, New Zealand; 3 Florida Museum of Natural History, University of Florida, Gainesville, Florida, United States of America; National Cheng Kung University, TAIWAN

## Abstract

Amphibians are the vertebrate group with the highest number of species threatened with extinction, and habitat loss and fragmentation are considered to be among the leading causes of their declines and extinctions. Little is known of the population biology of amphibian species inhabiting montane forests in Central and West Africa, where anthropogenic activities such as farming and cattle raising are major threats to native biodiversity. We used Amplified Fragment Length Polymorphisms (AFLPs) to assess the population genetic structure of two poorly known species, *Cardioglossa schioetzi* and *Leptodactylodon bicolor* (both in the Arthroleptidae), in and around Ngel Nyaki Forest Reserve on the Mambilla Plateau in eastern Nigeria. The landscape comprises continuous forest on steep slopes and small riparian forest fragments in a grassland matrix. While increased fragmentation is well documented for these and other forests in the mountains of Cameroon and Nigeria over the past century, there are no previous assessments of the impact of forest fragmentation on montane amphibian populations in this region. Our estimates of genetic diversity are similar across populations within each species with levels of heterozygosity values consistent with local population declines. Except for a pair of populations (*C*. *schioetzi*) we did not observe genetic differentiation between forest and riparian forest fragment populations, nor across sites within continuous forest (*L*. *bicolor*). Our results demonstrate recent gene flow between forest fragments and the adjacent protected forests and suggest that small forest corridors connecting these may lessen the genetic consequences of at least 30 years of intense and severe fragmentation in Ngel Nyaki.

## Introduction

Globally, amphibians face the most extreme population declines of all major vertebrate groups [1–3]. Contributing factors include climate change, disease, and habitat fragmentation [4–6]. The severity of amphibian declines vary across geographic regions [3] and despite evidence for population declines in Africa [7], the influence of habitat fragmentation on these declines remains unknown [7, 8]. Even less is known about the impact of habitat fragmentation on population genetics of amphibian species in Africa [9,10].

As is the case with many other taxa, habitat fragmentation likely has long-term effects on the genetic viability of amphibian populations because of the combined effects of reduced population size and increased isolation [11]. Increased isolation among populations may lead to reduced dispersal and gene flow, increased levels of inbreeding, smaller effective population sizes, and loss of genetic variation [12–14]. Consequently, assessing the population genetics of a species can provide valuable information for conservation much more quickly than longitudinal demographic studies [15]. Such studies have already been widely used to inform management strategies to halt or slow down amphibian decline [10,16–22].

To begin to redress the paucity of population genetics studies of African amphibians, we focused on populations of frogs within the forests of the Nigerian Highlands of the Cameroon Volcanic Line [23]. This is one of Africa’s biodiversity hotspots [24] and hosts a rich diversity of amphibian species [25]. Despite recognition as a center of biodiversity [26,27], the diversity and ecology of amphibians in Nigeria’s mountains remain understudied [25]. The montane forests on the Mambilla Plateau have been fragmented for a long time, hundreds of years at least. However, even 30 years ago there was more connectivity among the fragments [28] and more likely the streamside forests were acting as corridors between larger forests. Within the last 30 years (HC, personal observation) there has been a dramatic increase in cattle, which first arrived on the Plateau in the 1950's [29]. In addition to overgrazing by cattle, other threats to these forests include grass burning and land clearance for farming [30]. We used this heavily modified landscape in Nigeria’s mountains as a context for studying how forest fragmentation and associated degradation impact population genetic structure in forest-associated frog species.

**Fig 1:**
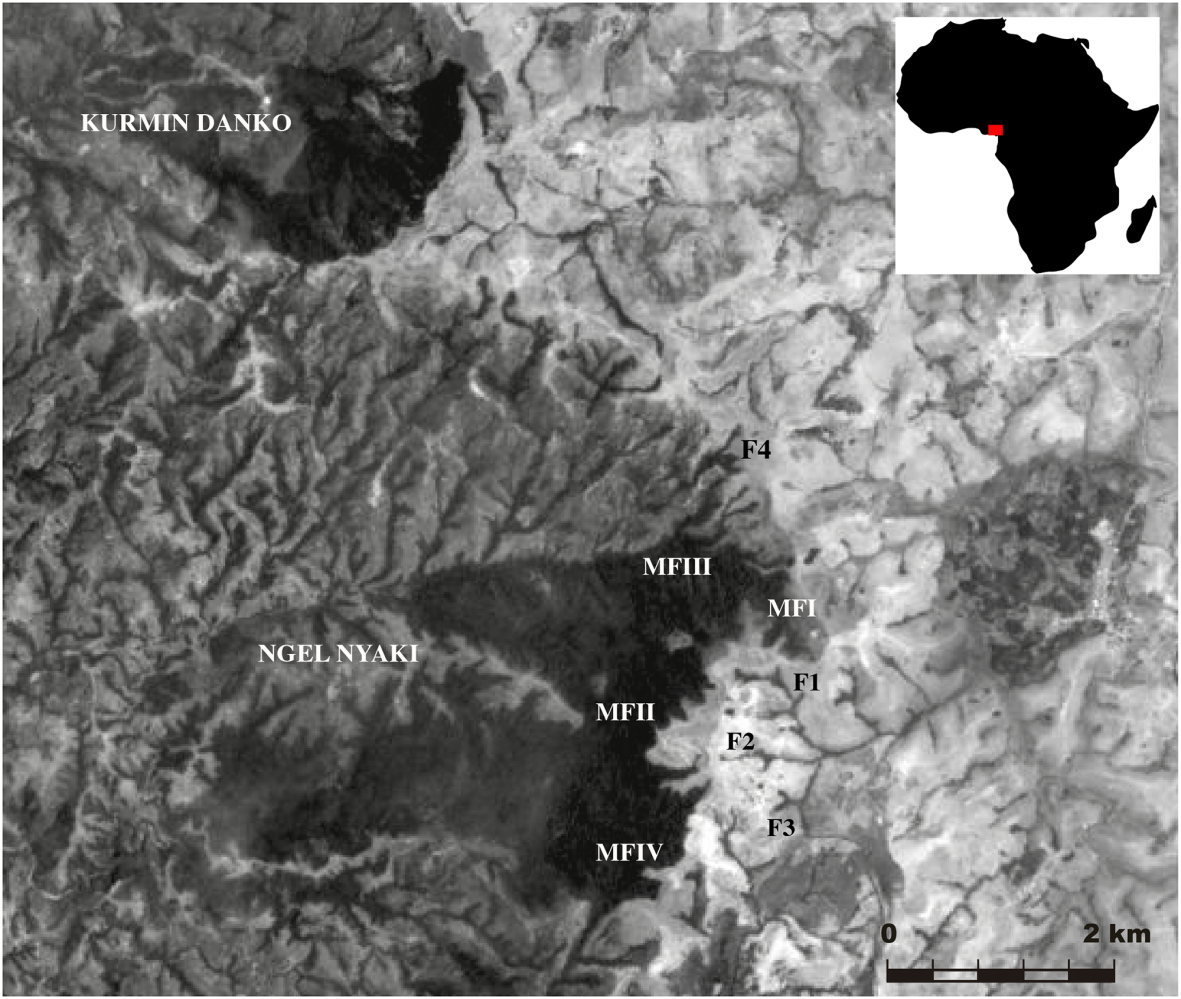
Sampling sites. Localities sampled for the frog species of interest in Ngel Nyaki at the Mambilla Plateau, Nigeria.

Based on previous surveys of Ngel Nyaki amphibians [25,31,32], we selected two species from the anuran family Arthroleptidae that is endemic to sub-Saharan Africa [33–37]. Both species, *Cardioglossa schioetzi* and *Leptodactylodon bicolor*, are endemic to the montane forests of Cameroon and Nigeria, they are small (<30 mm snout–vent length), have stream-adapted tadpoles, and live in leaf litter and rocky areas [37–39]. Little information exists on the population biology of these species, such as for example, the distances over which individuals disperse. *Cardioglossa schioetzi* occurs within small degraded riparian forests close to, but outside Ngel Nyaki Forest Reserve, as well as along the edge habitat of the continuous Ngel Nyaki forest (Fig 1). In contrast, *L*. *bicolor* is common along rocky streams within the continuous forest, but is rarely found within riparian fragments (Fig 1; [32]). Both species are on the IUCN Threatened species list (*C*. *schioetzi*–Endangered; *L*. *bicolor*–Vulnerable [40]) because of habitat degradation and the decline of remaining forest habitats on the mountains of Cameroon and Nigeria.

We aimed to determine levels of genetic diversity within and among populations of each species and use these data to assess levels of gene flow and connectivity among the populations within each species, in part to determine the extent to which grasslands act as a dispersal barrier for these species. Because its populations occur in fragments surrounded by heavily grazed grassland, we hypothesized that *C*. *schioetzi* would have less connectivity among populations and less genetic diversity than in *L*. *bicolor*.

## Material and methods

### Study area and field sampling

The Mambilla Plateau in Taraba State Nigeria (Fig 1) is located on the margins of the Cameroon Highlands Forest ecoregion that is well known for its rich flora and fauna, which are among the most diverse in Africa [41]. A high proportion of taxa are regional endemics [27,28]. Ngel Nyaki Forest Reserve (7°30’N, 11°30’E) is part of a network of sub-montane/montane forests and forest fragments at elevations up to 2300 m, with a mean annual rainfall of approximately 1800 mm and mean monthly temperatures of 13–26 °C and 16–23 °C for the wet and dry seasons, respectively [30]. The reserve, on the western escarpment of the Mambilla Plateau covers approximately 4600 ha and comprises a mosaic of overgrazed montane grasslands, degraded streamside forest/shrubland strips and 720 ha of dense sub-montane forest [28,30]. We refer to these riparian fragments as ‘fragments’ and Ngel Nyaki forest as ‘continuous forest’. The continuous forest has a rich floristic composition with over 146 vascular plant species, many of which are endemic to Afromontane regions, including four IUCN Red Data Listed species [28]. The fragments comprise a subset of the forest species and are typically more open and disturbed than the forest.

Between July and October 2012, we searched for *C*. *schioetzi* and *L*. *bicolor* in both forest and fragments (Fig 1, Table 1). We used a combination of visual and acoustic techniques [32] to find the frogs, searching for at least four hours during the day and another four hours by the night. After two weeks of searching, we identified sites with sufficient numbers of individuals to meet our minimum sample size of (N = 15) for characterizing a population [32]: four sites within the forest: Main Forest I (MFI), Main Forest II (MFII), Main Forest III (MFIII), and Main Forest IV (MFIV); and four fragments from outside of the reserve: Fragment 1 (F1), Fragment 2 (F2), Fragment 3 (F3), and Fragment 4 (F4). In total, we collected 69 adult toe clips and 11 tadpole tail tips for *C*. *schioetzi*, and for *L*. *bicolor* one toe clip and 117 tadpole tail tips. Tissue samples were preserved in microtubes with 95% ethanol and genetic analyses were performed at the University of Canterbury, New Zealand. This study was conducted with approval of the Animal Ethics Committee of the University of Canterbury (permit Ref: 2012/24R); the field permit was granted for field work in Ngel Nyaki Forest Reserve.

**Table 1:** Sample site information for *Cardioglossa schioetzi* and *Leptodactylodon bicolor*. Number of animals sampled/genotyped (N), Percentage of Polymorphic Loci (PPL), and Expected Heterozygosity (*H*_E_). The standard error (SE) is in brackets and was calculated over loci for each population, assuming each chromatogram peak represents a different locus.

Species	Locality Code	Latitude	Longitude	Elevation (m)	N	PPL	*H*_E_
*C*. *schioetzi*	MFI	7° 5.117'	11° 3.918'	1543	13	66.4	0.221(0.019)
	F1	7° 4.737'	11° 3.934'	1643	26	84	0.227 (0.016)
	F2	7° 4.939'	11° 3.218'	1647	20	71.20	0.199 (0.017)
	F3	7° 4.573'	11° 3.821'	1655	21	77.60	0.229 (0.016)
*L*. *bicolor*	MFI	7° 5.179'	11° 3.894'	1505	31	76.27	0.217(0.017)
	MFII	7° 4.859'	11° 3.465'	1641	20	65.25	0.198 (0.018)
	MFIII	7° 5.368'	11° 3.771'	1599	19	74.58	0.238 (0.018)
	MFIV	7° 4.498'	11° 3.282'	1561	25	66.10	0.208 (0.018)
	F4	7° 6.092'	11° 3.613'	1601	23	66.95	0.184 (0.017)

### AFLP profiling and data analysis

We chose to use AFLPs in our study because, while we are well aware of their limitaions [42] and the fact that hypervariable genetic markers would have been preferable [15,43], we are working on species without genomic resources to refer to (e.g. [18,44]). AFLPs have been shown to be a cost-effective and rapid tool for generating many polymorphic loci useful for inferring population genetic structure of species [45–47]. While we may not have achieved the precison we could have using more sensitive molecular markers, we believe our data to be important and a significant contribution to what is known about amphibian population genetic diversity in West Africa. Moreover, an added advantage of using AFLPs is that it allowed us to make direct comparisons with other African studies [18,44,46,48,49].

We extracted genomic DNA from tissue samples using a modified CTAB (cetyltrimethylammonium bromide) protocol [50] and developed AFLPs based on Vos et al. [51] with minor modifications. We used the two enzymes EcoRI and MseI to conduct digestion of genomic DNA. We then ligated fragments to double-stranded adaptors with T4 DNA ligase. For pre-selective PCR, we used primers complementary to the Eco RI with no added nucleotides and MseI+A. For the selective PCR, we used two pairs primers: ESP1B / MSP3 and ESP1B / MSP6 (Table 2). The EcoRI selective primer, ESP1B, was labelled with a fluorescent dye (either 6-FAM or VIC, Applied Biosystems) and contained three selective bases, whereas MSP3 and MSP6 (MseI selective primers) contained four bases of extension each (Table 2). We ran the selectively amplified fragments on an ABI 3130xl Genetic Analyzer (Applied Biosystems) with Gene-Scan LIZ size standard (Applied Biosystems). Finally, we visualized AFLP fragments with GENEMAPPER 4 (Applied Biosystems) in which peaks were called using the default settings except for the peak height detection which was set at 100 Relative Fluorescent Units (RFU). It was assumed that each peak represents a different locus. The threshold intensity for a peak being considered a locus was 100 RFU with a length at least 100 bp. Above 300 bp the resolution was such that it was not possible to accurately identify peaks, so that only peaks between 100-300bp were included in the analysis. The presence or absence of all fragments was confirmed manually. Following Stölting et al. [52] markers were scored for all the individuals in the same analysis session to prevent scoring errors when analyzing several groups of samples.

**Table 2:** Restriction enzymes, adapters and primers sequences used on the AFLP procedure. *Labeled with fluorescence.

		Sequence (5’-3’)
Restriction enzymes	EcoRI MseI	G^AATTC CTTAA^G T^TAA AAT^T
Adapters	EA2 EA3 MA1 MA2	CTCGTAGACTGCGTACC AATTGGTACGCAGTCTAC GACGATGAGTCCTGAG TACTCAGGACTCAT
Pre-selective primers	ENP MNP	GACTGCGTACCAATT GATGAGTCCTGAGTAA
Selective Primers	ESP1B MSP3 MSP6	GACTGCGTACCAATTCAG* GATGAGTCCTGAGTAACGAT GATGAGTCCTGAGTAACCTC

We conducted reproducibility tests by replicating samples, as suggested by Bonin et al. [53] and Meudt and Clarke [54]. As recommended, we replicated analyses for a subset of samples (ideally 5–10% of the total number of samples) to detect fragments producing erratic patterns. To do so, we randomly selected 5% of the samples for each preferred primer combination for duplication and then assessed the error rate.

Due to the fact that mitochondrial 16S DNA sequences of selected individuals from Ngel Nyaki for both *C*. *schioetzi* and *L*. *bicolor* are less than 2% divergent from populations on Mount Oku in the Cameroonian mountains (unpublished data), similar to *Arthroleptis palava* [31], we are confident that genetic differences observed on the Mambilla Plateau are due to population-level differences rather than cryptic species.

#### Genetic diversity

We used GenAlEx v6.5 [55] to perform frequency and distance-based analyses. Allelic frequencies were estimated following the method of Lynch and Milligan [56], assuming independent nuclear loci and Hardy–Weinberg equilibrium within populations. The expected Heterozygosity (*H*_E_) was calculated as 2(p)(q) implemented for diploid binary data (dominant markers) and assuming random mating, where q = (1—Band Frequency)^0.5 and p = 1—q.

We converted genetic data into a pairwise individual-by-individual genetic distance matrix and used this to assess genetic structure within populations. This is a true Euclidean metric [see 55] as required for the subsequent analysis of molecular variance.

#### Evaluating the population genetic structure

We performed an Analysis of Molecular Variance (AMOVA) to investigate the hierarchical partitioning of genetic variation among populations and estimate the population genetic differentiation via Φ_*PT*_. To do this, each of the sampling sites were treated as populations (other hierarchical analysis such as different groups of fragments or regions were not performed). Φ_*PT*_ is analogous to *F*_*ST*_ and used for dominant markers such as AFLP [57]. This measure was calculated as *V*_*AP*_ ⁄ (*V*_*AP*_ + *V*_*WP*_), where *V*_*AP*_ is the variance among populations and *V*_*WP*_ the variance within populations. We tested for statistical significance of the Φ_*PT*_ values using a nonparametric permutation method [58] with the number of permutations set to 999. By testing significance of the variance components and Φ statistic through a permutational approach removes the assumption of normality, which is unsuitable for molecular data [58]. Significance levels following corrections for multiple comparisons via Bonferroni tests [59] were conducted.

Last, we investigated population genetic structure using the Bayesian model-based clustering algorithms implemented in STRUCTURE v2.3.3 [60]. STRUCTURE uses a Markov chain Monte Carlo (MCMC) algorithm to cluster individuals into populations based on their genotypes. It generates posterior probabilities of assignment of individuals to each of a given number of *K* groups or populations regardless of their site of origin [60]. For each species, we first ran STRUCTURE on all populations across the entire study area using the admixture model (no prior information) with correlated allele frquencies. We then used the population data (sampling sites) as prior information (LOCPRIOR) to assist the clustering as recommended when the signal of structure is weak [60,61]. For both species, a range of *K* values from 1 to 10 was performed. We ran batches of five independent runs with a burn-in of 100,000 and 500,000 iterations for each of the *K* values. We calculated the ‘optimal’ *K* (the number of genetic clusters that best fits these data) using the web version of STRUCTURE HARVESTER [62]. Below, we report estimates for *K* using two methods: (1) the log posterior probability of the data Ln(*K*) given *K* genetic clusters [60] and (2) Δ*K* [63], which is based on the rate of change in the log probability of data between successive *K* values.

To test for migration within each species, we used STRUCTURE to identify immigrants or individuals with recent immigrant ancestry in the last two generations. The model for this analysis uses population information with correlated allele frequencies. The model was set to GENSBACK = 2 and MIGRPRIOR = 0.001, as suggested by Falush et al. [64]. Thus, the prior probability that an individual has pure ancestry from its predefined population is 0.999.

To examine the pattern of isolation by distance, we tested for correlation between matrices of geographic and genetic distances (Φ_*PT*_ pairwise_)_ using a Mantel test as implemented in GenAlEx v6.5.

Although both GenAlEx and STRUCTURE can accommodate missing data, we removed missing genotypes from the analyses for the two preferred combinations of primers for both species in all analyses. Missing data may be problematic for some pairwise distance-based analyses implemented in GenAlEx [65]. While GenAlEx provides an option for interpolating the genetic distance for missing loci (by inserting the average genetic distances for each population level pairwise contrast, that is, within a population, or between two populations), we prevent any possible bias by eliminating the missing data from our analyses [see 55].

## Results

The two species had different distributions. *Cardioglossa schioetzi* was recorded most often near streams in riparian forests outside the boundary of Ngel Nyaki Forest Reserve and sometimes on the edge of the continuous forest within the reserve. Specimens of this species were common in F1, but rare in F2 and F3 (see Fig 1). In contrast, *L*. *bicolor* was common along the streams within the forest, but encountered in only one riparian forest outside of the reserve.

The two preferred primer combinations, ESP1B/MSP3 and ESP1B/MSP6, together yielded 275 loci for the 198 samples representing both species (S1 Table). Of these 275 loci, 243 (88.3%) were polymorphic. We estimated the genotyping error by comparing the presence-absence matrices of repeated profiles. The genotyping error rate per locus was calculated as the ratio between the number of single-locus mismatches (*ml*) and the number of replicated loci (*nt*) [66]. The estimated genotyping error per locus was 2.9% (SD = 3.9) for ESP1B/MSP3 and 5.2% (SD = 4.3) for ESP1B/MSP6. We obtained an average error rate of 4% by taking into account the two pairs of primers.

### Genetic diversity

Similar values of genetic diversity were obtained for both the four populations of *C*. *schioetzi* and the five populations of *L*. *bicolor* (Table 1). The average percentage of polymorphic loci for the four populations of *C*. *schioetzi* was 74.8% (Standard Error SE = 3.8). The average of genetic diversity (*H*_E_) across these populations and loci was 0.219 (SE = 0.008). We did not detect significant differences in average heterozygosity between populations (F(3,496) = 0.65, P = 0.58). The average percentage of polymorphic loci for *L*. *bicolor* was 69.8% (SE = 2.3), and the average value of genetic diversity was *H*_E_ = 0.209 (SE = 0.008). Average heterozygosity was not significantly different among the five populations of *L*. *bicolor* (F (4,585) = 1.28, P = 0.28).

### Population structure

According to the overall Φ_*PT*_ (calculated via AMOVA) no significant genetic differentiation was detected among the populations of *C*. *schioetzi* (Φ_*PT*_ = 0.018, p = 0.097). After Bonferroni correction, pairwise estimates of Φ_PT_ among populations (Table 3) showed differences only among the population from the forest MFI with the riparian fragments F3. In contrast, no significant difference was recorded among the three riparian fragments. For *L*. *bicolor* no genetic differences were detected among the five populations following either the overall Φ_*PT*_ (Φ_*PT*_ = 0.026, p = 0.020) or the Φ_*PT*_ pairwise matrix (Table 4).

**Table 3:** Pairwise matrix for *Cardioglossa schioetzi* of Φ_PT_. Φ_PT_ values below diagonal, and above it probability values (significant values in bold, Bonferroni correction).

	MF1	F1	F2	F3
MF1	-	0.026	0.042	**0.005**
F1	0.051	-	0.424	0.398
F2	0.061	0.000	-	0.371
F3	0.101	0.000	0.000	-

**Table 4:** Pairwise matrix for *Leptodactylodon bicolor* of Φ_PT_. Φ_PT_ values below diagonal, and above it probability values.

	MFI	MFII	MFIII	MFIV	F4
MFI	-	0.103	0.260	0.280	0.013
MFII	0.021	-	0.112	0.366	0.007
MFIII	0.007	0.029	-	0.224	0.103
MFIV	0.003	0.001	0.011	-	0.055
F4	0.054	0.073	0.027	0.039	-

**Fig 2:**
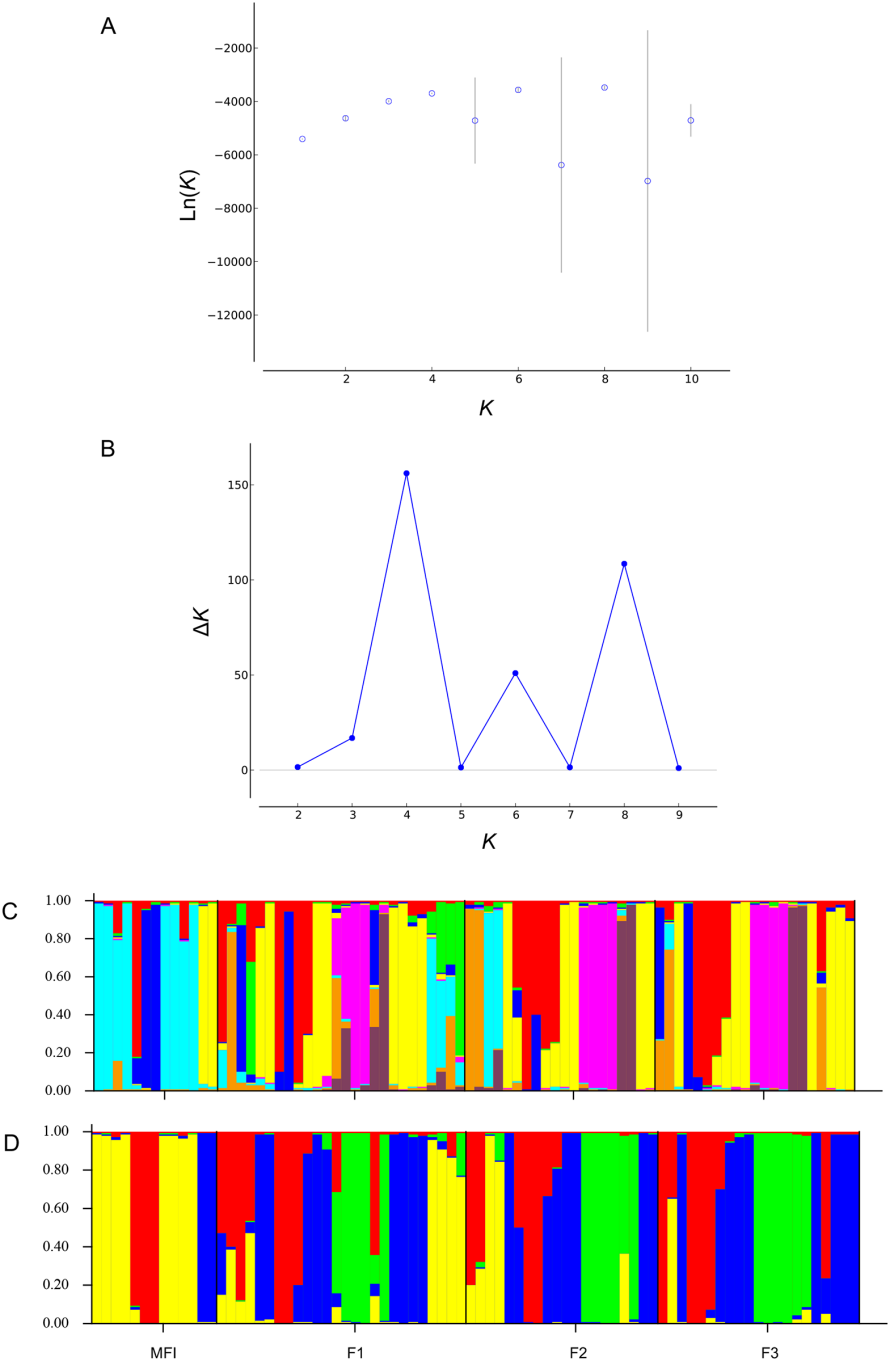
Structure assignments for *Cardioglossa schioetzi* using sampling location information. (A) *K* = 8 clusters based on the Ln(*K*), and (B) following Evanno’s method shows the assignment into Δ*K* = 4 clusters. The bar plots at the bottom show (C) *K* = 8 and (D) ΔK = 4. Each vertical bar represents an individual for which is shown the proportional genetic assignment.

**Fig 3:**
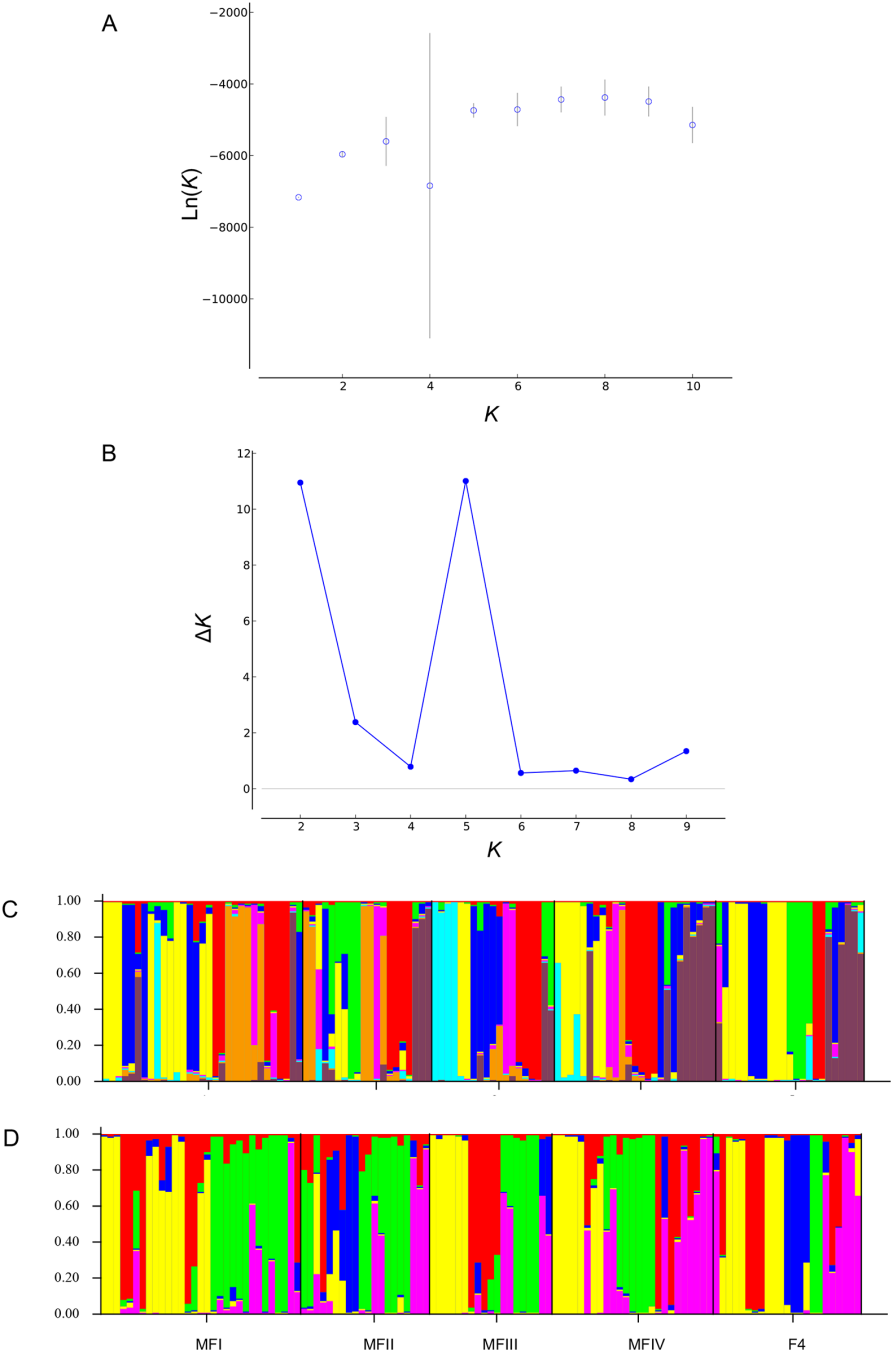
Structure estimates of *K* groups for *Leptodactylodon bicolor* using sampling location information. Graph (A) and bar plot (C) show *K* = 8 based on the Ln(*K*) estimation. (B) and (D) represent respectively the graph and plot of the best *K* following Evanno’s approach Δ*K* = 5. Each vertical bar represents an individual for which is shown the proportional genetic assignment.

The clustering analyses implemented in STRUCTURE (both with and without prior information) revealed multiple groups for both species. For *C*. *schioetzi*, the analysis using all the sites across the study area without prior location information (S1 Fig) found an optimal *K* (based on the Ln(*K*)) of 5, and Δ*K* = 2 based on Evanno’s method. When using prior information (Fig 2), *K* = 8 and Δ*K* = 4 were recognized as the best *K* for each of the methods, respectively. For *L*. *bicolor*, without prior location information (S2 Fig), the optimal *K* (based on the Ln(*K*)) was 7, and Δ*K* = 2. Based on the Ln(*K*) estimation, with prior location information, eight genetic groups (*K* = 8) were detected, whereas the Evanno’s estimation detected five clusters (Δ*K* = 5) (Fig 3).

### Migration test

The test for migration revealed that 53 of 80 individuals of *C*. *schioetzi* were immigrants or had ancestry in other populations in the past two generations. Migration between forest and riparian fragments was 40% (21 individuals) and between riparian fragments was 60% (32 individuals). In the case of *L*. *bicolor*, 78 of 118 (66%) were immigrants or had ancestry in other populations in the past two generations. Twenty-one (27%) individuals of *L*. *bicolor* were migrants between forest and riparian fragments, whereas 57 (73%) were between the populations within the continuous forest. Due to the fact that the tissue samples used in this study were toe clips from males (*Cardioglossa schioetzi*) and tadpole tails (*Leptodactylodon bicolor*), it was not possible to test if migration is biased by sex.

### Genetic isolation

No significant correlation was observed in either species between geographic and genetic distance (using Φ_*PT*_; *C*. *schioetzi* r = 0.226, P = 0.432; *L*. *bicolor* r = 0.511, P = 0.134).

## Discussion

Our study aim was to investigate the extent to which fragmentation of existing forests may impact gene flow and connectivity among populations of two Afromontane anuran species. While Afromontane forests are typically small and naturally fragmented relative to lowland forests [67,68], the extent and rate of fragmentation on the Mambilla Plateau has increased since the 1970’s in response to increasing human and cattle populations [30]. We detected low levels of genetic variation and genetic differentiation among forest and fragment populations of both *C*. *schioetzi* and *L*. *bicolor*. However, the two species differed somewhat in patterns of genetic diversity and gene flow among populations, which may reflect differences in dependency on forest.

### Within-population genetic variation

We estimated genetic diversity based on the proportion of polymorphic loci and expected heterozygosity; measures which are commonly used in studies based on dominant markers such as AFLP’s. This allowed us to directly compare our results with previous AFLP studies on amphibians undergoing local population declines. We found that our observed levels of genetic diversity for both *C*. *schioetzi* and *L*. *bicolor* are within the range reported from such previous studies (Table 5). For example, Curtis and Taylor [69] evaluated the impact of forest clearcuts on the population structure of *Dicamptodon tenebrosus* in southwestern British Columbia and reported heterozygosity (*H*_E_) ranging from 0.192 to 0.285 in recently clearcut sites.

**Table 5:** Heterozygosity values reported for AFLP on amphibians undergoing local population declines.

Species	Sample size	Heterozygosity range	Source
*Cardioglossa schioetzi*	80	0.199–0.229	This study
*Leptodactylodon bicolor*	118	0.184–0.238	This study
*Amietia wittei*	180	0.228–0.294	Zancolli et al. 2014
*Amietia angolensis*	301	0.223–0.343	Zancolli et al. 2014
*Arthroleptis xenodactyloides*	141	0.223–0.324	Measey et al. 2007
*Gastrophryne carolinesis*	100	0.165	Makowsky et al. 2009
*Calotriton asper*	241	0.006–0.105	Mila et al. 2010
*Eurycea nana*	85	0.161–0.180	Lucas et al. 2009

### Genetic differentiation and genetic structure of populations

A common cause of genetic differentiation among populations is geographic distance so that in continuous habitats, poor dispersal ability and large distances between individuals may drive genetic differentiation [70,71]. However, the results of our Mantel test on the continuous forest populations of *L*. *bicolor* showed no evidence for significant genetic differences among them, regardless of distance. Within the continuous forest habitat dispersal occurs over at least 1.85 km (between MFIII and MFIV), the greatest distances between any of our continuous forest sites. The fact that estimates of *F*_*ST*_ from AFLP’s are likely to be over estimates [72,73] gives us confidence that little, if any genetic differentiation exists among forest populations.

Habitat fragmentation, which often isolates populations and reduces their size [72] is another cause of population genetic differentiation at small geographic scales, e.g. less than five km [72,73]. We found evidence for this in *C*. *schioetzi*, where a population from the riparian forest fragment (F3) is distinct from the forest population (MFI). While F3 and MFI are in close geographic proximity (less than two km), they are separated by heavily overgrazed and annually burnt grassland, so that movement between habitats would likely be difficult. However, because AFLPs have a lower mutation rate than microsatellites [74,75] it is important to be aware that our estimates of *F*_*ST*_ will likely be higher than if we had used microsatellites [69,75].

Similar findings have been reported by Dixo et al. [76] who compared genetic diversity of the toad *Rhinella ornata* among small and medium forest fragments that were either isolated or connected to large forest areas by corridors. They found a weak but significant *F*_*ST*_ between small fragments and continuous forest, but no significant genetic differentiation among continuous forest sites.

The extent of gene flow among local populations determines their potential for genetic differentiation [77]. Thus, at our study site, current gene flow may explain the lack of genetic differentiation among populations within the forest (*L*. *bicolor*) and among the riparian forest fragments (*C*. *schioetzi*). This is supported by our tests for migration, which indicated that in both species, populations are weakly linked by gene flow, both current and within the past two generations. In the case of *C*. *schioetzi*, the test revealed that population F3 contained immigrants from the other two riparian fragments as well as from the forest population MFI. In this case, invidivuals may be dispersing via a stepping-stone dispersal model [78], moving, for example, from MFI to F3 through F1, most probably in the rainy season when grass is tall and there is no burning. Moreover, at this time of year streamflow is higher so that streams within fragments which are isolated from each other in the dry season are able to join up, connecting fragments (see Fig 1). Despite this potential homogenizing effect of the wet season, signiticant genetic difference was detected among some population pairs. In the case of *L*. *bicolor*, the single riparian fragment population F4, contained immigrants from all forest populations except MFII, from which it was significantly genetically different. A possible explanation for this could be non-random gene flow, i.e. gene flow which is not constant over time, nor the same level between every population pair. Such variation could reduce the homogenizing effect of gene flow and instead promote genetic differentiation [79,80]. Two studies we are aware of on *Parus major* have demonstrated that non-random dispersal (such as we describe above) can contribute to genetic differentiation at a fine scale [79,80]. For example Garant et al. [79] found that non random dispersal influenced diversifying effects on body mass variation and Postma and van Noordwijk [80] observed that small-scale genetic difference in clutch size was produced by different levels of gene flow.

For both species, the results of clustering analyses in STRUCTURE differ based on the estimator used (Ln(*K*) and Δ*K*) and groupings were not consistent with sampling location. While the precise number of genetic clusters is not critical to our study, the finding of multiple population groups using both estimators supports our interpretation of population genetic structure.

A synthesis of our results suggest they are similar to the findings of a similar study on *Phrynobatrachus guineensis* in Taï National Park (TNP), Ivory Coast [81]. As in our study, and in contrast to expectations, Sandberger et al. [81] detected only a slight significant genetic differentiation among populations of this leaf litter frog and no correlation between the geographic and genetic distances (isolation by distance). Their Bayesian clustering revealed no genetic substructure. Originally *P*. *guineensis* was thought to be weakly mobile and highly specialized, however, high intra- and possibly inter-patch migration events explained the lack of population structure. Thus, individuals of this species are more able to disperse than was expected. We cannot rule out that this is not the case in our study and future work is needed to test this idea.

Another potential contributing factor to genetic differentaion in fragmented populations is that in fragmented landscapes species often persist as metapopulations [82]. This may well be the case for the two target species in this study [83]. If so, then the persistence of genetic variation within and among populations would depend on the factors discussed above and the ability of the species to form a metapopulation [84].

While we lack information on dispersal distances for our focal species, some examples are available for other amphibian species. For the salamander *Plethodon cinereus*, Cabe et al. [85] found clear genetic differences among plots that increased with distance (200m to 2 km). Geographic distances at larger scales (> 2 km) contributed to differentiation among populations of the European tree frog *Hyla arborea* in a fragmented landscape Angelone et al. [86]. Because the likelihood of detecting isolation by distance increases with the number of populations sampled [87], the lack of correlation in our study between geographic distance and genetic differentiation may be due to the relatively small number of sample sites included. However, sampling more sites at greater distances is difficult in this system as Ngel Nyaki Forest Reserve comprises only ~5.3 km^2^ of forest (with no straight-line distances within continuous forest exceeding 5 km) and most of the few remaining forest fragments are relatively near to the reserve.

### Implications for conservation

Our results contribute to the relatively limited body of knowledge of dispersal in modified landscapes for African amphibians [e.g., 18,81]. Our results suggest that despite considerable habitat degradation (especially in the riparian fragments) gene flow is still occurring (or occurred recently) among forest patches in two Afromontane frog species. This illustrates the importance of these degraded riparian forest fragments to amphibian communities.

In Nigeria and all along the Cameroon Volcanic Line [88,89], as elsewhere in the tropics [90,91], the survival of riparian forest fragments is under threat. Often fragments are not given the official protection garnered by larger patches of continuous forest [92] with disasterous consequences [93]. Evidence that these fragments not only harbour biodiversity but also have on-going gene-flow with neighboring continuous forest may provide added leverage to conservation practitioners aiming to protect isolated populations in forest fragments. On the Mambilla Plateau of eatern Nigeria, conservation efforts should focus not only on the existing Ngel Nyaki and Kurmin Danko Forest Reserves but also the small riparian forest fragments on the periphery of these forests as well as the many more like them across the mountains of Cameroon and Nigeria.

## Supporting information

S1 Fig Outcomes from structure without using population data for *Cardioglossa schioetzi*. Graph A) and bar plot C) depict the optimal *K* based on the Ln(*K*) *K* = 5, whereas graph B) and bar plot D) show the better *K* based on Evanno‘s method Δ*K* = 2. Each vertical bar represents an individual for which is shown the proportional genetic assignment to each cluster.
(TIF)

S2 Fig Outcomes from structure without using population data for *Leptodactylodon bicolor*. Graph (A) and bar plot (C) depict the optimal *K* based on the Ln(*K*) *K* = 7, whereas graph (B) and bar plot (D) show the better *K* based on Evanno‘s method Δ*K* = 2. Each vertical bar represents an individual for which is shown the proportional genetic assignment to each cluster.
(TIF)

S1 Table Raw data of *Cardioglossa schioetzi* and *Leptodactylodon bicolor*. Together, the two preferred primer combinations ESP1B/MSP3 and ESP1B/MSP6 yielded 275 loci for the 198 samples representing both species.
(XLSX)
