# Supplementary figures and images for: Conservation genetics of two threatened frogs from the Mambilla highlands, Nigeria
Source: PLoS One. 2018 Aug 15;13(8):e0202010. doi: 10.1371/journal.pone.0202010 (PMC6093670; doi:10.1371/journal.pone.0202010)

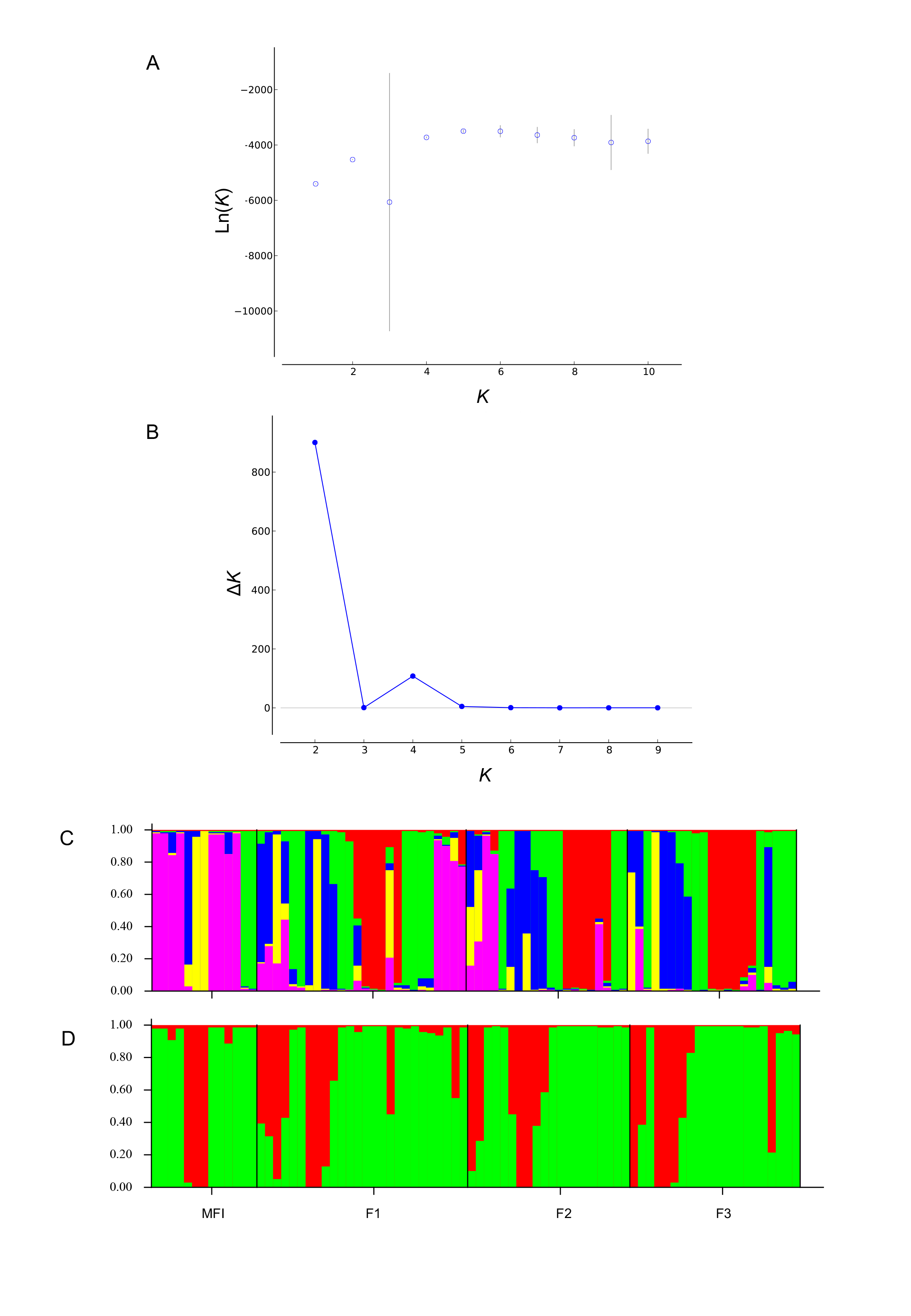

Supplement: S1 Fig — Graph A) and bar plot C) depict the optimal K based on the Ln(K) K = 5, whereas graph B) and bar plot D) show the better K based on Evanno‘s method ΔK = 2. Each vertical bar represents an individual for which is shown the proportional genetic assignment to each cluster. (TIF) [file pone.0202010.s001.tif]

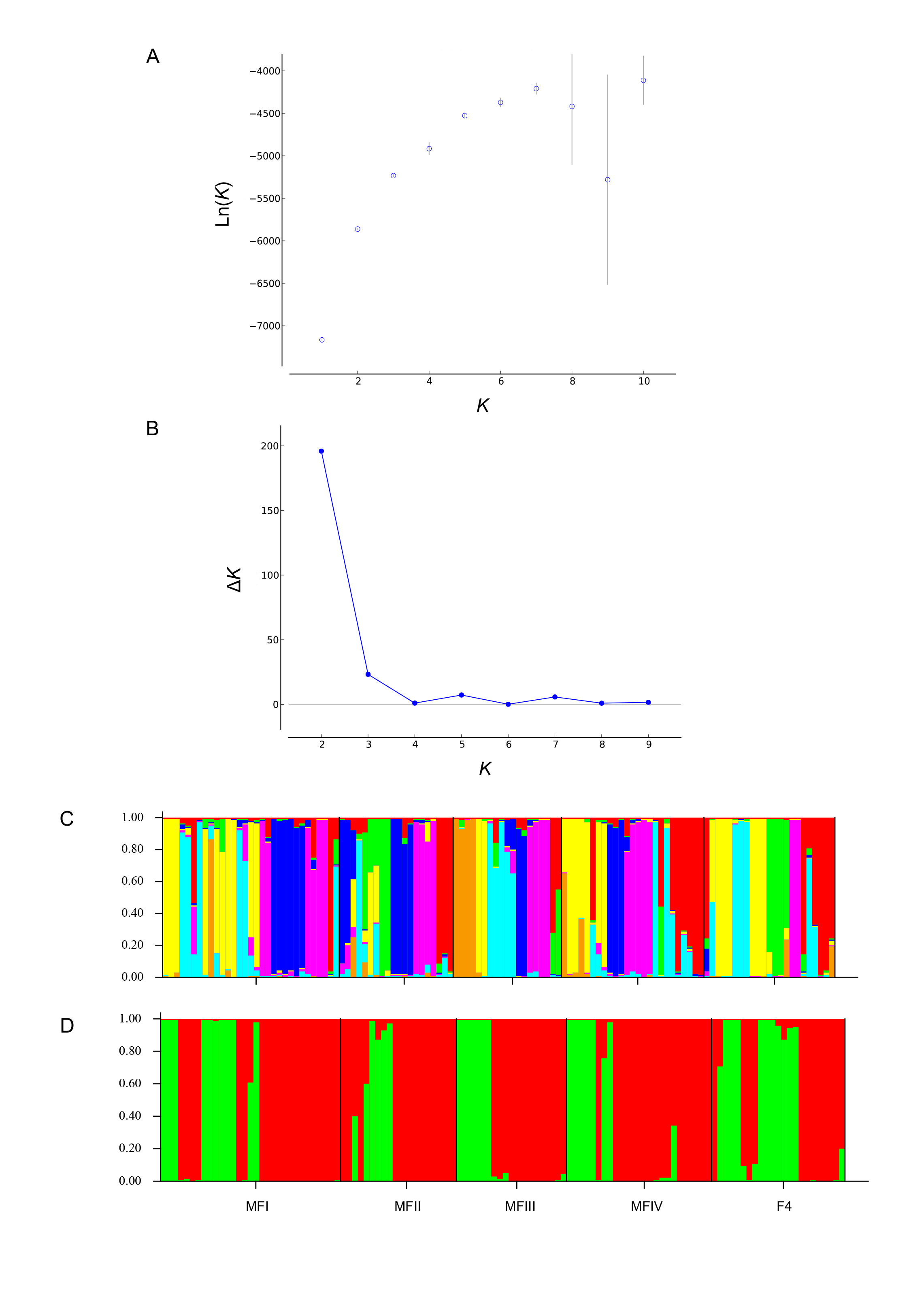

Supplement: S2 Fig — Graph (A) and bar plot (C) depict the optimal K based on the Ln(K) K = 7, whereas graph (B) and bar plot (D) show the better K based on Evanno‘s method ΔK = 2. Each vertical bar represents an individual for which is shown the proportional genetic assignment to each cluster. (TIF) [file pone.0202010.s002.tif]
